# The Role of Oxytocin Neurons in the Paraventricular Nucleus in Chronic-Sleep-Deprivation-Mediated Abnormal Cardiovascular Responses

**DOI:** 10.3390/cimb47040220

**Published:** 2025-03-25

**Authors:** Yifei Zhang, Yuxin Wang, Zhendong Xu, Xiangjie Kong, Hairong Wang, Zhibing Lu, Ming Chen, Linlin Bi

**Affiliations:** 1 Department of Pathology, Taikang Medical School (School of Basic Medical Sciences), Wuhan University, Wuhan 430071, China; 2022203010046@whu.edu.cn (Y.Z.); yuxinwang@whu.edu.cn (Y.W.); 2 Center for Pathology and Molecular Diagnostics, Zhongnan Hospital of Wuhan University, Wuhan University, Wuhan 430071, China; 3 Department of Cardiology, Zhongnan Hospital of Wuhan University, Wuhan University, Wuhan 430071, China; xzd6699@163.com (Z.X.); xiangjiekong1989@163.com (X.K.); wanghai2192@sina.com (H.W.); luzhibing222@163.com (Z.L.); 4 Institute of Myocardial Injury and Repair, Wuhan University, Wuhan 430071, China; 5 Hubei Provincial Clinical Research Center for Cardiovascular Intervention, Wuhan 430071, China; 6 Guangdong Province Key Laboratory of Psychiatric Disorders, Southern Medical University, Guangzhou 510515, China

**Keywords:** chronic sleep deprivation, oxytocin neurons, paraventricular nucleus, blood pressure, heart rates, 3-mercaptopyruvate sulfurtransferase

## Abstract

Sleep disorders increase the risk of cardiovascular diseases. However, the underlying mechanisms remain unclear. This study aims to examine the critical role of oxytocin neurons in the paraventricular nucleus (PVN^OXT^) in regulating the cardiovascular system and to elucidate potential mechanisms through which sleep disturbance may contribute to cardiovascular diseases. In this study, using an automated sleep deprivation system, mice were given chronic sleep deprivation (cSD) for 7 days, 6 h per day. cSD induced blood transcriptomic alterations accompanied by lower heart rate, higher blood pressure, and elevated cardiac autophagy/apoptosis. Instant optogenetic activation of oxytocin neurons in the paraventricular nucleus (PVN^OXT^) provoked heart rate suppression in normal mice, whereas in cSD mice, activation precipitated intermittent cardiac arrest. On the contrary, inhibition of PVN^OXT^ showed no influence on the cardiovascular system of normal mice, but it attenuated cSD-induced rise in blood pressure. Long-term low-frequency stimulation (LTF) of PVN^OXT^ decreased neuronal excitability and oxytocin release, effectively reversing cSD-mediated cardiovascular responses. Mechanistically, cSD triggered the upregulation of blood-derived 3-mercaptopyruvate sulfurtransferase (mPST), and a suppression of PVN^OXT^ postsynaptic activity to a certain extent. The quick and long-term decrease of oxytocin by LTF could lead to feedback inhibition in mPST expression and thus reverse cSD-mediated cardiovascular responses. Altogether, modulation of PVN^OXT^ could mediate cSD-induced cardiovascular abnormalities without affecting normal mice. Our research provided potential targets and key mechanisms for cardiovascular diseases associated with sleep disorders.

## 1. Introduction

Reduced risks of cardiovascular diseases are associated with healthy sleep patterns [1,2,3,4,5]. Sleep disorders, such as excessive lethargy, late chronotype, and insomnia, are associated with a 10–40% increased risk of cardiovascular disease [1,6,7,8]. Some previous studies suggested that sleep disruption after myocardial infarction promoted oxidative stress and NO production and aggravated the enlargement of the heart, which might ultimately lead to cardiac dysfunction [9,10]. A higher risk of hypertension is linked to partial sleep deprivation, which also impairs photoplethysmography and heart rate variability [11,12]. Thus, elucidating the pathophysiological links between sleep deprivation and cardiovascular damage may unveil novel therapeutic targets for intervention.

The hypothalamic-synthesized nonapeptide oxytocin, a neurohormone critical for parturition and mammary gland function in mammals, originates predominantly from magnocellular neurons within the paraventricular and supraoptic nuclei [13]. The oxytocin system might be related to cardiovascular homeostasis [14,15,16,17,18,19]. In the magnocellular portion of the paraventricular nucleus (PVN), oxytocin-containing magnocellular neurons project to the neurohypophysis, where they are discharged into the hypophyseal portal and systemic circulation. In the rat, the vasculature synthesized oxytocin neurotransmitter and its receptors [20]. The G protein-coupled receptor family includes oxytocin receptors, which can increase intracellular calcium by activating phospholipase C and mitogen-activated protein kinase [21]. There has been some controversy regarding the research on whether or how oxytocin is involved in regulating the cardiovascular system [14]. Over-expression of OXT receptors within hypothalamic paraventricular nuclei enhanced arterial baroreflex sensitivity and buffer blood pressure in unanesthetized rodents. [22]. Pretreatment of rats with selective blockade of oxytocin receptors increased heart rate variability during stress [22]. Both central and peripheral injections of oxytocin could affect blood pressure, but the responses were variable [14,23]. Nevertheless, whether the endogenous oxytocin was essential for the maintenance of blood pressure and heart rate in normal mice was unknown. Our understanding of the underlying mechanisms connecting cardiovascular disease to sleep was limited. Whether oxytocin neurons were involved in sleep-deprivation-mediated abnormal cardiovascular responses was also unclear.

The PVN is essential for regulating the cardiovascular system. The PVN includes neuroendocrine (neurosecretory) and autonomic compartments, both of which are important for regulating osmotic homeostasis and blood pressure [19,24,25]. In the PVN, neurosecretory magnocellular neurons are the primary source of oxytocin [24]. Additionally, neurosecretory parvocellular neurons have a role in stress responses and the functioning of the hypothalamic–pituitary–adrenal axis [24,26]. Furthermore, the PVN, an autonomic master controller of the cardiovascular system, is essential for the autonomic adaptation of blood pressure to external stressors and causes blood pressure variability [24]. Chronic stimulation of hypothalamic oxytocin neurons attenuated the development of hypertension following an additional four weeks of chronic intermittent hypoxia exposure (CIH) [27], indicating that PVN contributed to CIH-induced exaggerated cardiovascular responses [28,29,30,31]. In hypertensive animals exposed to CIH, activation of PVN oxytocin neurons decreased heart rate but facilitated faster heart rate recovery time following exercise. In the CIH model, decreased heart rates induced by the oxytocin neurons could help animals reduce oxygen consumption, which thus played a protective role in the cardiovascular system. However, the PVN oxytocin’s role in the cardiovascular system following cSD is not yet fully elucidated.

As a key enzymatic source of hydrogen sulfide in cardiovascular and neurological systems, 3-mercaptopyruvate sulfurtransferase (mPST) plays a crucial regulatory role in maintaining cardiovascular homeostasis [32,33]. Previous studies have found that mPST-knockout mice showed changed brain function [32,34,35], while dual ablation of cystathionine gamma lyase and mPST (CTH/mPST) demonstrated enhanced vasodilation and hypotensive effects [33]. Notably, the therapeutic potential of oxytocin–H2S interaction has emerged in addressing trauma-related physiological and psychological sequelae, particularly for mitigating acute post-traumatic complications in chronic medical conditions [36]. Previous studies also found that sleep deprivation could impair the blood–brain barrier [37] and induce systemic inflammation, leading to neuroinflammation by cytokine transport across the impaired blood–brain barrier [38,39]. Whether mPST could enter the brain through the damaged blood–brain barrier was unknown. Whether oxytocin and mPST interact following chronic sleep deprivation was also unclear.

By employing cell-selective neuromodulation strategies, we conducted an optogenetic method of PVN^OXT^ to verify their pathophysiological contribution to chronic-sleep-restriction-induced cardiovascular dysregulation. We also investigated the relevant factors in the blood transcriptome and the autophagy and apoptosis of cardiac tissue associated with cSD, especially the mPST. Our research aimed to discover the complex roles of oxytocin neurons in regulating cardiovascular responses following cSD. We tried to provide potential explanations and targets for abnormal cardiovascular system changes elicited by cSD. Our investigation extended to optogenetic modulation strategies aimed at regulating synaptic dynamics. This methodological exploration provides translational insights for cardiovascular therapeutics, particularly through deep brain stimulation applications that promote targeted neuroplasticity in specific neural circuits.

## 2. Materials and Methods

### 2.1. Animals

All protocols complied strictly with National Research Council guidelines for the care and use of laboratory animals. Male C57BL/6J (Hunan SJA Laboratory Animal Co., Ltd., Changsha, China) mice (8–12 weeks old, weighing 25–30 g) were randomly selected and maintained under standardized conditions: room temperature 22 ± 2 °C, relative humidity 50–60%, with ad libitum access to food/water and a 12:12 h light/dark cycle (zeitgeber time 0 initialization). All behavioral tests were performed during the light cycle between 9:00 and 17:00. All experimental procedures were performed and approved by the Wuhan University Institute of Animal Welfare (Approval number AF031, 2020-04-03).

### 2.2. Virus Injection and In Vivo Optogenetic Stimulation

Mice (8–12 weeks old) were first anesthetized via intraperitoneal injection (i.p.) of sodium pentobarbital (50 mg/kg) (RWD Life Technology Co., Ltd., Shenzhen, China) and positioned on a stereotaxic device. Ophthalmic ointment was administered to the mouse’s eyes to prevent corneal dryness and eye damage. Next, the scalp was shaved and cleaned with iodine and 70% alcohol, and then a longitudinal incision was made along the midline to reveal the skull. With the aid of a microscope, tiny holes were drilled above the PVN (AP: −0.6 mm, ML: ±0.2 mm, DV: −4.8 mm) for viral injections (RWD Life Technology Co., Ltd., Shenzhen, China). The virus was injected at a rate of 10 nl/min using a micropipette fitted with an Auto-Nanoliter Injector (Harvard).

For optogenetic manipulation of PVN-OXT neurons, PVN was injected with AAV2/9-OXT-mCherry, AAV2/9-OXT-ChR2-mCherry, or AAV2/9-OXT-eNpHR3.0-mCherry (50 nl for each microinjection, brain VTA according to the Franklin and Paxinos Mouse Brain Atlas). For OXT neurotransmitter probe virus in mPFC, the virus (rAAV9-hSyn-OT1.8; 2 × 10^12^ genomic copies per ml in mixtures) or a control virus (AAV2/9-DIO-Ef1a-EYFP; 3.9 × 10^12^ genomic copies per ml in mixtures) was injected (all viruses packaged by Brain Case Co., Ltd., Wuhan, Shenzhen, China). Two weeks after the virus injection, a fiber ferrule (Inper Tech, Shenzhen, China) was implanted 200 μm above the PVN (AP: −0.6 mm, ML: ±0.2 mm, DV: −4.6 mm) or mPFC (AP: 1.95 mm, ML: ±0.25 mm, DV: −2.10 mm). Behavioral tests were carried out one week after the fiber ferrule implantation. Any cases when viral expression occurred outside of the PVN area were omitted from the analysis. Photostimulation parameters were calibrated using a Thorlabs PM100D optical meter, with ChR2 activation achieved through 5–8 mW 473 nm laser delivery at fiber termini. PVN^OXT^ neuronal excitation employed a stochastic stimulation paradigm (20 Hz, 10 ms pulse width) via blue-light illumination. Concurrently, eNpHR3.0-mediated optical inhibition protocols implemented 589 nm wavelength light within PVN microcircuits.

### 2.3. Oxytocin Receptor Antagonist

Mice received oxytocin antagonist L-368899 or atosiban 1 h before all behavioral tests. Injections were administered intraperitoneally in all experiments (1 mg/kg/100 μL; Tocris Bioscience, Bristol, UK) according to the previous studies [40,41,42,43].

### 2.4. mPST Inhibitor

The cell-permeable mPST inhibitor I3MT-3 (HY-128206, MecChem Express, Monmouth Junction, NJ, USA) working solution preparation was as follows: 1 mL aliquot of the dosing solution was prepared by mixing 100 μL of clear DMSO stock solution (20.8 mg/mL) with 900 μL of corn oil vehicle and homogeneous mixed into the final solution. This formulation produced a solution with ≥2.08 mg/mL drug concentration. The final dosage concentration administered was 16 mg/kg. Vehicle control groups received equivalent volumes of DMSO–corn oil mixture without active compound.

### 2.5. Sleep Deprivation

Using an automated sleep deprivation system, which consisted of an automatic cylinder (PVC cylinder, height: 60 cm, width: 50 cm, weight: 5 kg, Rifeng Enterprise Group Co., Ltd., Foshan, China), a small slow motor, and a metal bar (45 cm) connected to the small motor, mice were subjected to 6 h of sleep deprivation (from 3 p.m.–9 p.m.) from day 1 to day 7. To induce sleep deprivation, the bar was continuously rotated at a speed of approximately 3 rpm and randomly reversed in the direction of rotation to prevent subjects from gaining short sleep periods by adapting to the rotation pattern. A trained experimenter visually verified that the wooden bar was spinning during the sleep deprivation to ensure that mice had no chance to sleep.

### 2.6. Echocardiography

Echocardiography was conducted as previously described [44]. A small animal ultrasound imaging system, the VINNO D860 LAB (Beijing Yeeran technology Co., Ltd., Beijing, China), was used to perform echocardiography. To measure the morphological and functional parameters, mice were anesthetized with 1.5% isoflurane, and long-axis and short-axis echocardiograms were obtained. M-mode measurements provided information on left ventricular internal diameter at diastole (LVIDd) and systole (LVIDs). The EF and FS data were measured using onboard ultrasound analysis software.

### 2.7. Blood Pressure and Heart Rate Monitoring

A Millar catheter transducer (1.4-French, SPR-839) was inserted into the left ventricular cavity through the carotid artery, and blood pressure and heart rate were captured and analyzed by PVAN software V2.3 (Small animal ultrasound imaging system VINNO D860 LAB, Beijing Yeeran Technology Co., Ltd., Beijing, China) as previously described [44].

### 2.8. Histology

After administering 50 mg/kg i.p. intraperitoneal sodium pentobarbital to sedate the mice, 30 mL of ice-cold saline (0.9% sodium chloride) and 50 mL of ice-cold 4% paraformaldehyde in PBS were infused transcardially. After that, mouse brains were removed from the skull and postfixed for an additional night in 4% paraformaldehyde. They were then dehydrated in PBS in 30% sucrose for 48 h at 4 °C. Brain tissues were sectioned into slices that were 30 μm thick using a microtome (CM 1900, Leica). For c-fos or OXT neuron staining, sections were soaked in a blocking solution (Beyotime), incubated for 20 min at room temperature (22 ± 2 °C), and rinsed three times in PBS for 10 min each. Next, either the rabbit polyclonal anti-oxytocin-neurophysin antibody (1:500, ab212193, ABcam, Waltham, MA, USA) or the rabbit polyclonal anti-C-fos antibody (1:10,000, ABE457, Millipore, Burlington, MA, USA) was used. The sections were incubated for 24 h with an anti-rabbit c-fos antibody or an anti-rabbit oxytocin-neurophysin antibody in a blocking solution at 4 °C. Then, three more PBS washes were performed (10 s each). Following primary antibody treatment, tissue specimens underwent 90 min incubation with species-matched IgG conjugates: Thermo Scientific Alexa Flour 488 donkey anti-rabbit IgG (1:500). Without c-fos staining, sections were washed 3 times with PBS, directly stained with DAPI, mounted onto glass microscope slides, dried, and coated with mounting material. The images of sections were taken with an Olympus BX53 fluorescent microscope (BX53, Olympus Corporation, Tokyo, Japan) and processed with ImageJ 1.52V software.

### 2.9. RNA Isolation and RNA-Seq Library Construction

Pharmacological anesthesia was induced in mice via intraperitoneal administration of pentobarbital sodium (50 mg/kg). Following orbital surgery, 1 mL of blood was collected in EDTA anticoagulation tubes (TIANAI). Three mice were sampled in each group, and RNA was extracted from the mice’s blood. RNAiso Plus (TAKARA) was used to isolate RNA according to the manufacturer’s instructions. All RNA-seq raw fastq data were provided by Wekemo Tech Group Co., Ltd. Shenzhen, China.

### 2.10. Western Blot Assay (WB)

Western blot analysis was performed as previously reported [45]. Tissue was extracted using RIPA lysis buffer with the protease inhibitor phenylmethane sulfonyl fluoride (Beyotime Biotechnology, Beijing, China). According to the manufacturer’s instructions, protein concentrations were measured using the BCA protein assay kit (Beyotime Biotechnology, Shanghai, China). RIPA lysis buffer was used to equalize the protein concentrations. Samples were denatured in 5× Laemmli buffer (4% SDS, 16% glycerol, 20% β-mercaptoethanol, 0.05% bromophenol blue) via 5 min boiling, followed by ultralow-temperature preservation (−80 °C) prior to electrophoretic separation.

Equivalent amounts of protein (20 μg) were separated on SDS-polyacrylamide gel electrophoresis gels. After incubation in blocking buffer (5% bovine serum albumin in Tris-buffered saline with Tween-20 (TBST)) at room temperature for 1 h, the membranes were incubated with primary antibodies (Rabbit SQSTM1/p62 Antibody, CST, catalog no. 5114; Rabbit Monoclonal LC3B(D11)XP@ Antibody, CST, catalog no. 3868; Rabbit Cleaved Caspase-3(Asp175)(5A1E) Antibody, CST, catalog no. 9664; Rabbit Bax Antibody, CST, catalog no. 2772; Mouse BCL2 Antibody, BIOLIGHT, catalog no. MA00081HM10-C2F; anti-his tag antibody, Sigma-Aldrich, catalog no. SAB5600227; Rabbit Monoclonal GAPDH Antibody, MedChemExpress, catalog no. HY-P80137; anti-β-actin antibody, ZSGB-BIO, Beijing, China, catalog no. TA09) at 4 °C overnight. After three washes in TBST, the membranes were incubated with a secondary antibody (goat anti-rabbit or mouse IgG, Santa Cruz Biotechnology, Dallas, TX, USA, 1:5000 dilution in blocking buffer) for 1 h at indoor temperature. Following triplicate washing cycles (5 min each) using TBST under the same temperature conditions, proteins were detected using ECL reagent (Millipore). Protein quantification was conducted through chemiluminescent detection employing the Bio-Rad ChemiDoc MP imaging platform (Hercules, CA, USA). Subsequent densitometric analysis of electrophoretic bands was performed with Bio-Rad’s Quantity One software suite (v4.4.0) following manufacturer-recommended protocols. To ensure statistical rigor, data points demonstrating significant deviation from population distributions underwent systematic exclusion prior to final data interpretation.

### 2.11. Electrophysiological Analysis

Pharmacological induction of anesthesia in rodent subjects was achieved through intraperitoneal administration of pentobarbital sodium (50 mg/kg), and their brains were rapidly removed and chilled in an artificial cerebrospinal fluid (ACSF) containing 220 mM sucrose, 1.3 mM CaCl_2_, 2.5 mM KCl, 1 mM NaH_2_PO_4_, 2.5 mM MgSO_4_, 10 mM glucose, and 26 mM NaHCO3. Brain tissues (300 μm) were sectioned using a VT-1000S vibratome (Leica, Wetzlar, Germany) and incubated at 34 °C with normal ACSF (in mM, 126 NaCl, 1 MgSO_4_, 3 KCl, 1.25 NaH_2_PO_4_, 2 CaCl_2_, 10 glucose, and 26 NaHCO_3_) to recover for 30 min, followed by an additional 1 h at 25 °C before recording. All solutions were saturated with 95% O_2_/5% CO_2_ (vol/vol). At 32–34 °C, regular ACSF was super-fused into the recording chamber at 2 mL/min. Neurons were recorded using pClamp 9.2 software (Axon Instruments, Scottsdale, AZ, USA) and whole-cell voltage-clamp methods (MultiClamp 700B amplifier, Digidata 1320A analog-to-digital converter). The following solution was filled into the glass pipettes: 105 mMK-gluconate, 30 mM KCl, 10 mM HEPES, 10 mM phosphocreatine, 4 mM ATP-Mg, 0.3 mM GTP-Na, 0.3 mM EGTA, and 5 mM QX314 (pH 7.35, 285 mOsm). EPSCs were recorded in the presence of the GABAAR antagonist BMI (20 μM). The resistance of pipettes was 3–5 MΩ. When evoked synaptic currents were created, a two-concentric bipolar stimulating electrode was placed about 100 μm from the recorded neuron. Stimulation intensity was standardized to evoke 50% maximal neuronal response amplitude. Electrical stimulation parameters comprised 0.2 ms pulses at 0.1 Hz frequency, synchronized through an A.M.P.I Mater-8 programmable stimulator. Neuronal recordings employed a −70 mV holding potential for excitatory postsynaptic current (eEPSC) measurements. Experimental recordings proceeded only when access resistance variation remained below 15% of baseline (initial range: 8–15 MΩ).

### 2.12. Data Analysis

The number of experimental animals was indicated by “n”. Every data set satisfied the normalcy assumption. To make paired comparisons, analyses employed independent *t*-test or two-way ANOVA followed by a repeated-measure ANOVA. When equivalent variances were assumed for numerous comparisons, the SNK test was utilized; otherwise, Dunnett’s test was employed. SPSS software (SPSS 26.0, Inc., Chicago, IL, USA) was used to conduct statistical analyses throughout the study. All data were expressed as mean ± SEM. *p* < 0.05 indicates significant values.

## 3. Results

### 3.1. Chronic Sleep Deprivation (cSD) Decreased the Heart Rate, Increased Blood Pressure, Changed the Blood Transcriptome, and Enhanced the Autophagy and Apoptosis of Cardiac Tissue

**Figure 1:**
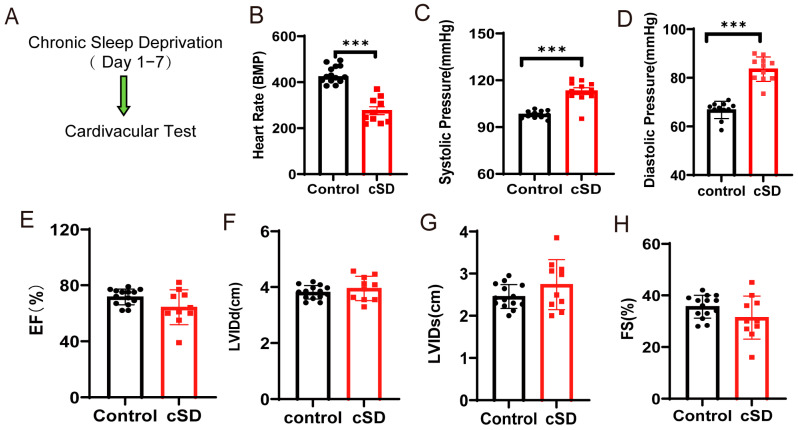

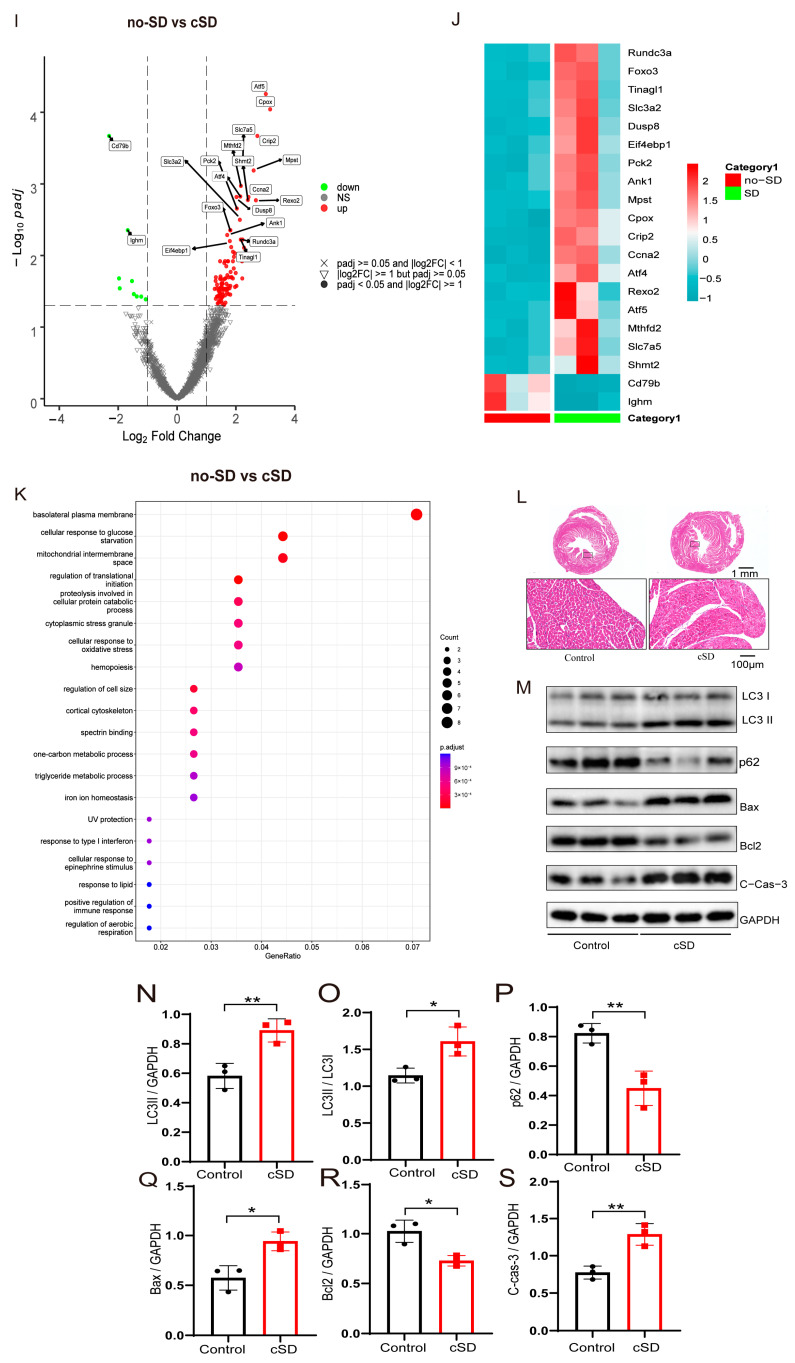
Chronic sleep deprivation (cSD) decreased the heart rate, increased blood pressure, changed the blood transcriptome, and enhanced the autophagy and apoptosis of cardiac tissue. (**A**) A schematic diagram of the experiment. (**B**) Comparison of the heart rates of mice between the control and cSD groups. (**C**,**D**) Blood pressure was measured through the carotid artery in vivo using an animal flowmeter. A comparison of the systolic and diastolic pressure of mice between the control and cSD groups is displayed. The comparisons of EF (**E**), LVIDd (**F**), LVIDs (**G**), and FS (**H**) between the control and cSD groups were separately calculated. The heart rates, EF, LVIDd, LVIDs, and FS were measured using echocardiography. (**I**) Volcano plots showing differentially expressed genes (DEGs) in comparisons of blood from no-SD and SD mice. Vertical dashed lines denote fold-change thresholds (|log2FC| = 1); the horizontal dashed line denotes the *p* adjust threshold (*p* < 0.05). (**J**) Heat map showing fold changes of top 20 genes between no-SD group and SD group. The color red represents a high level of gene expression, while the color blue indicates a low level of gene expression. (**K**) GO analysis of differentially expressed genes (DEGs) in SD compared with no-SD. The top 20 ranked GO biological processes, molecular functions (MFs), and cellular components (CCs) are shown. (**L**) Hematoxylin and eosin (H&E) images of crosscut section of the heart from control and cSD mice (scale bar = 1 mm or 100 μm). (**M**–**S**) Representative western blot image and quantification of LC3-I, LC3-II, p62, Bax, Bcl2, and c-Cas-3 SOD2 in control and cSD mouse hearts (n = 3/group). For 1B–H, the significance of the difference between groups was determined using independent *t*-test (control, n = 14, cSD, n = 10). Vertical bars represent the mean ± the SEM. Asterisks indicate significant differences from the relevant controls, (* *p* < 0.05, ** *p* < 0.01, *** *p* < 0.001, two-tailed *t*-test for (**P**–**S**)).

To assess whether cSD affected the heart rate, blood pressure, and left ventricular function, we subjected the mice to 7 days of cSD. Following the cSD, echocardiography was used to evaluate the heart rates, ejection fraction (EF), fractional shortening (FS), left ventricular internal diameter at systole (LVIDs), and left ventricular internal diameter at diastole (LVIDd) (Figure 1A). The blood pressure was measured through the carotid artery in vivo using an animal flowmeter. We found that the 7 days of sleep deprivation significantly decreased the heart rates (t(22) = 8.459, *p* < 0.0001; Figure 1B), increased the systolic pressures and diastolic pressures (t(22) = 6.547 or 9.063, both *p* < 0.0001; Figure 1C,D). However, the cSD did not affect the cardiac systolic function of mice. Neither the EF, nor LVIDd, nor LVIDs, nor FS were different between the control and the cSD group (Figure 1E–H).

To identify the molecular factors underlying the change in blood pressure and heart rates induced by the cSD, both no-SD and SD mice were subjected to RNA-seq analyses. We interrogated transcriptional changes in the adult mice’s blood after chronic sleep deprivation. Genes with *p* adjust < 0.05 and fold change ≥ 1 were regarded as differentially expressed genes (DEGs). Seven days of cSD induced extensive alterations to the blood transcriptome in comparison to no-SD controls, as illustrated by the heatmap of the fold-changes between the SD group and the no-SD group (Figure 1I,J). In male mice, this resulted in 114 genes that were differentially expressed (105 upregulated and 9 downregulated). The genes that we found to be significantly upregulated due to chronic sleep deprivation were Atf5, mPST, Mthfd2, Slc7a5, Shmt2, and Rexo2 (Figure 1J). Collectively, cSD increased genes involved in cellular metabolism (Mthfd2, Slc7a5, and Shmt2) and maintenance of mitochondrial homeostasis (Atf5 and Rexo2). Chronic sleep deprivation downregulated the level of immune-related genes (Cd79b and lghm). We next performed a functional analysis for each differentially expressed gene (Figure 1K) and found that these genes were mainly enriched in biological functions such as cell structure and catabolism. Furthermore, compared to the control groups (no-SD), the functional annotation network illustrated the biological and molecular roles of pathways modified by cSD. These results indicated that cSD enhanced the inflammatory and cellular stress responses.

We next tested whether the autophagy and apoptosis of cardiac tissue were associated with cSD. As shown in Figure 1L, myocardial cells were arranged neatly, with dense cytoplasm and clear contour boundaries in the normal group of mice. However, cSD altered the morphological structure of myocardial cells, leading to disordered arrangement, less dense cytoplasm with granular degeneration, and unclear contour boundaries. Compared to the control group, the expression of microtubule-associated protein-1 light chain 3-II (LC3-II), the autophagy marker, and autophagic flux indicator LC3-II/LC3-I ratio was increased by cSD (t(4) = 4.619, *p* = 0.0099; t(4) = 3.617, *p* = 0.0224; Figure 1M–O). Conversely, the expression of p62, an autophagy adaptor protein that accumulated when autophagy was inhibited, was decreased (t(4) = 4.811, *p* = 0.0086; Figure 1M,P). cSD also increased the expression of apoptotic protein Bax (t(4) = 4.113, *p* = 0.0147; Figure 1M,Q) and cleaved casepase-3 (t(4) = 5.227, *p* = 0.0064; Figure 1M,S), and remarkably reduced Bcl-2 (t(4) = 4.141, *p* = 0.0144; Figure 1M,R). These data indicated that cSD induced excessive autophagy and apoptosis in the heart.

### 3.2. Optogenetic Activation of PVN^OXT^ Neurons of Normal Mice Decreased Heart Rates Without Affecting the Left Ventricular Function and Blood Pressure

**Figure 2:**
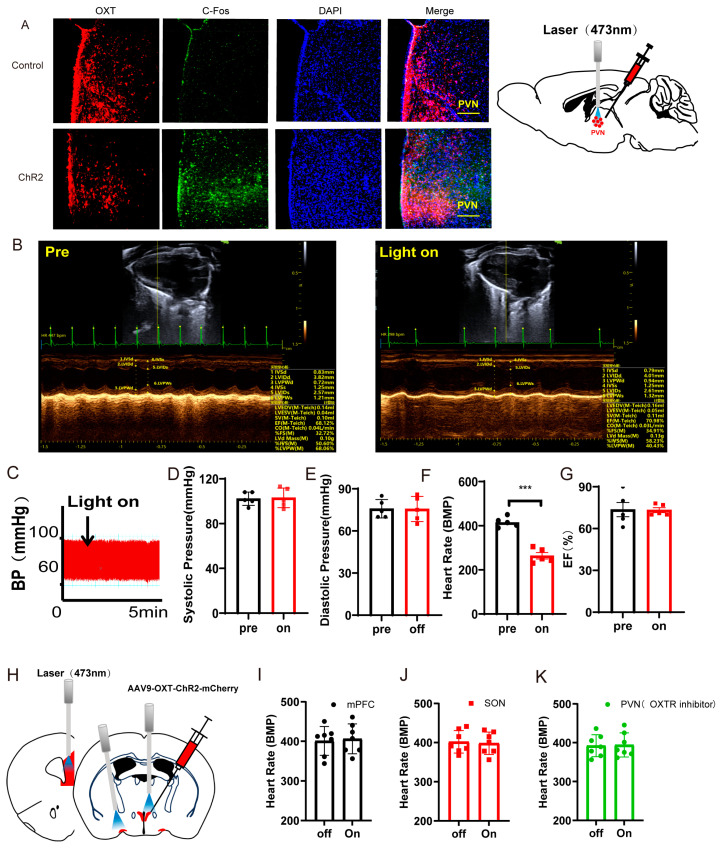
Optogenetic activation of PVN^OXT^ neurons in normal mice decreased heart rates. (**A**) Representative immunohistochemical staining pictures of AAV-OXT-hChR2-mCherry viral injection (left panel) and optogenetic stimulation of OXT neurons with 473 nm laser (right panel). AAV-OXT-ChR2-mCherry expression (red), c-Fos (green), and DAPI (blue) in the PVN are shown (scale bar = 200 μm). (**B**) Echocardiography results from mice before and during the light-on phase. (**C**) The blood pressure was measured from mice before and during the light-on phase. (**D**) The average systolic pressure before and after the light was measured. (**E**) The average diastolic pressure before and after the light was analyzed. (**F**) The heart rates before and after the light were analyzed. (**G**) The EF values before and after the light were measured. (**H**) AAV9-OXT-mCherry virus was injected into the PVN area. The optical fiber was implanted into the mPFC, the SON, and the PVN. (**I**,**J**) The heart rates before and after the light were analyzed (n = 7/group). (**K**) For the PVN group, an OXTR inhibitor (atosiban) was injected intraperitoneally into mice. The heart rates before and after the light were analyzed (n = 7/group). Statistical significance was determined using the two-way RM ANOVA. The significance of the difference between groups was determined using a paired *t*-test (n = 5/group, *** *p* < 0.001).

According to earlier research, PVN^OXT^ neurons may have a role in controlling the cardiovascular system [19]. Next, we looked into how heart rate, blood pressure, and left ventricular function were affected by optogenetic activation of PVN^OXT^ neurons. Representative immunohistochemical staining pictures showed that the AAV-OXT-hChR2-mCherry virus was injected into the PVN (Figure 2A, left panel). Optogenetic stimulation of OXT neurons was carried with a 473 nm laser (Figure 2A, right panel). Optogenetic activation of PVN^OXT^ neurons increased c-Fos expression in AAV-OXT-ChR2-mCherry-expressing neurons, which proved the effectiveness of the virus (Figure 2A). To further determine the specificity of AAV9-OXT-ChR2-mCherry expression in the oxytocin neurons, we injected the AAV-AVP-hChR2-mCherry virus into the PVN. Unlike the functions of oxytocin neurons, activation of arginine vasopressin neurons did not affect the heart rates but increased both the systolic and diastolic pressure, which indirectly proved the specificity of the AAV9-OXT-ChR2-mCherry virus (Figure S1).

Echocardiography results and blood pressures recorded from mice before and during the light-on phase are shown in Figure 2B,C. Statistical analysis results showed that optogenetic activation of the PVN^OXT^ neurons could significantly decrease heart rates (t(5) = 23.56, *p* < 0.001, Figure 2F). However, activation of the PVN^OXT^ neurons had no effect on the systolic pressure, diastolic pressure, or EF (Figure 2D,E,G). The cSD decreased heart rates significantly, and optogenetic activation of PVN^OXT^-ChR2 neurons with 20 Hz blue light seriously decreased the heart rates after the cSD, with some subjects showing intermittent cardiac arrest (see Supplementary Video S1 data).

To test whether PVN^OXT^ neurons regulated the cardiovascular system through the release of oxytocin, we also measured the effect of activating the PVN^OXT^-mPFC circuit or the PVN^OXT^-SON circuit on heart rates. We found that optogenetic activation of the PVN^OXT^-mPFC pathway or the PVN^OXT^-SON pathway could not affect heart rates (Figure 2I,J and Figure S2). Though optogenetic activation of the PVN^OXT^ neurons could decrease the heart rate, it could not affect the heart rate in the presence of the OXT receptor inhibitor atosiban (which cannot pass the blood–brain barrier, Figure 2K). These results suggested that the PVN^OXT^ neurons might regulate the cardiovascular system through the OXT receptors in the peripheral cardiovascular system.

### 3.3. Instant Optogenetic Inhibition of PVN^OXT^ Neurons Could Reverse the Change of Blood Pressure Induced by the cSD but Not the Change of Heart Rates

The above results suggested that PVN^OXT^ neurons might be able to regulate the cSD-induced changes in the cardiovascular system. We first checked whether endogenous PVN^OXT^ neurons participated in maintaining blood pressure and heart rate in normal mice. Representative pictures showed that the AAV-OXT-eNpHR-mCherry virus was injected into the PVN (Figure S3A, left panel). Optogenetic inhibition of OXT neurons was carried out with a 589 nm laser (Figure S3A, right panel). We found that optogenetic inhibition of the PVN^OXT^ neurons had no effect on the heart rates, systolic pressures, diastolic pressures, or EF values of normal mice without cSD treatment (all *p* > 0.05, Figure S3).

**Figure 3:**
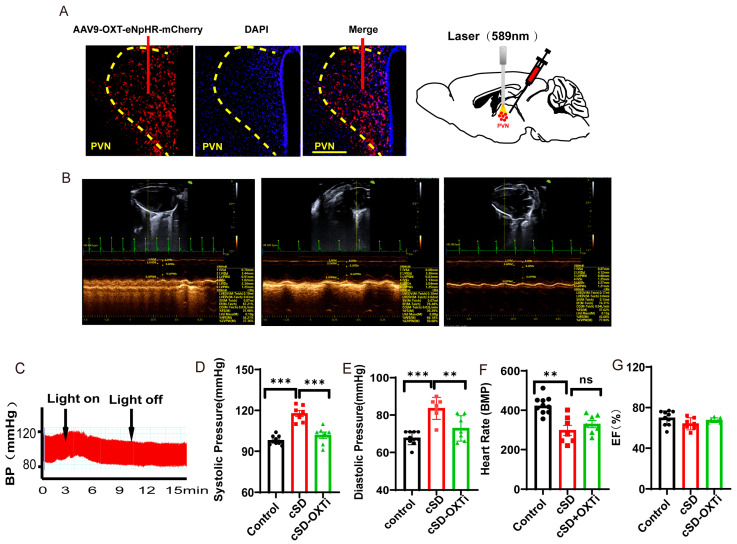
Instant optogenetic inhibition of PVN^OXT^ neurons could reverse the change of blood pressures induced by the cSD but not the change of heart rates. (**A**) Representative immunohistochemical staining pictures of AAV-OXT-eNpHR-mCherry virus injection (left panel) and optogenetic inhibition of OXT neurons with 589 nm laser (right panel). AAV-OXT-eNpHR-mCherry expression (red) and DAPI staining (blue) in the PVN are shown. (**B**) Echocardiography results of the control group, cSD group, and cSD + OXTi (inhibition of OXT neurons) are shown. (**C**) The representative blood pressure of cSD mice before and during the light-on phase is shown. (**D**) The averaged systolic pressures of control mice and cSD mice before and after the light were measured. (**E**) The averaged diastolic pressures of control mice and cSD mice before and after the light were analyzed. (**F**) The heart rates of control mice and cSD mice before and after the light were analyzed. (**G**) The EF values of control mice and cSD mice before and after the light were measured. The arrows indicate that the blue-light stimulation was delivered into the PVN of the ChR2 group during testing time. The significance of the difference between groups was determined using two-way ANOVA for C-G (control group, n = 10; cSD group, n = 7; and cSD + OXTi, n = 7). All error bars are s.e.m. (ns *p* > 0.05, ** *p* < 0.01, *** *p* < 0.001).

We then tested the effect of the optogenetic inhibition of PVN^OXT^ neurons on the cardiovascular system after the cSD. Representative pictures showed that the AAV-OXT-eNpHR-mCherry virus was injected into the PVN (Figure 3A, left panel). Optogenetic inhibition of OXT neurons was carried out with a 589 nm laser (Figure 3A, right panel). Echocardiography results and blood pressures recorded from mice of the control group, cSD group, and cSD + OXTi (optogenetic inhibition of OXT neurons) group (Figure 3B) and the representative blood pressure of cSD mice before and during the light-on phase (Figure 3C) are shown. Three weeks after the virus injection, mice were subjected to sleep deprivation for 7 days. After this, we found that the cSD increased the systolic pressures and diastolic pressures (F (2, 12) = 38.01 or F (2, 12) = 24.74; both *p* < 0.001; Figure 3D,E). The cSD decreased the heart rates but did not affect the EF values (F (2, 12) = 8.027, F (2, 12) = 2.082; *p* = 0.006, *p* = 0.166; Figure 3F,G). Optogenetic inhibition of PVN^OXT^ neurons could immediately reverse the effect of cSD on blood pressure (F (2, 12) = 38.01 or F (2, 12) = 24.74; *p* < 0.001, *p* = 0.002; Figure 3C–E), but did not affect the heart rates of cSD mice (F(2, 12) = 8.027; *p* = 0.561; Figure 3F). No significant differences in EF values were observed among all groups (F (2, 12) = 2.082, *p* = 0.1674; Figure 3G).

We speculated that the PVN^OXT^ neurons did not participate in the maintaining of blood pressure and heart rates in the normal condition but might be involved in regulating blood pressure and heart rates after the cSD. The effective onset time of inhibition of the PVN^OXT^ neurons for the heart rates and blood pressure after the cSD might be different. Short-time inhibition of PVN^OXT^ neurons could quickly restore blood pressure to normal after the cSD but could not affect heart rates.

### 3.4. Long-Term Low-Frequencies (LTF) Stimulation of PVN^OXT^ Neurons Could Reverse the Change in Blood Pressures, Heart Rates, the Transcriptome of the Blood, the Morphological Structure of Myocardial Cells, and the Autophagy and Apoptosis of Cardiac Tissue Induced by the cSD

**Figure 4:**
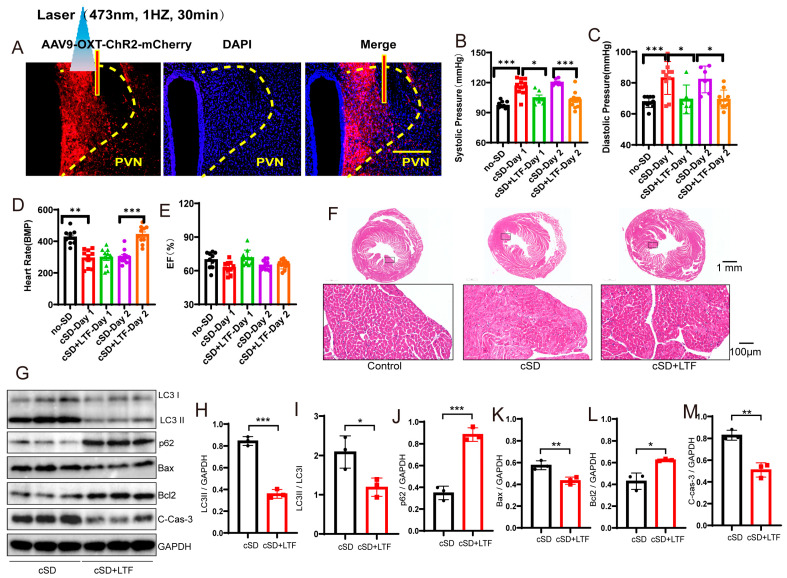

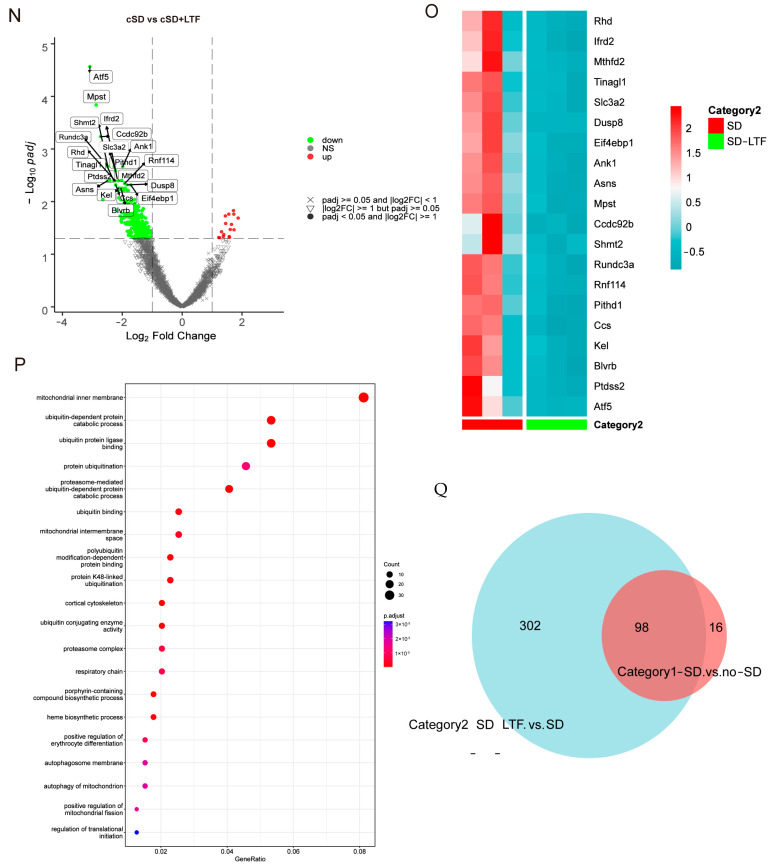
Long-term low-frequencies (LTF) stimulation of PVN^OXT^ neurons could reverse the change in blood pressures, heart rates, transcriptome of the blood, morphological structure of myocardial cells, and the autophagy and apoptosis of cardiac tissue induced by the cSD. (**A**) Representative immunohistochemical staining pictures of AAV-OXT-ChR2-mCherry viral injection (left panel) and optogenetic inhibition of OXT neurons with 473 nm laser (right panel). AAV-OXT-ChR2-mCherry expression (red) and DAPI staining (blue) in the PVN are shown. (**B**) The averaged systolic pressures were measured. (**C**) The averaged diastolic pressures were analyzed. (**D**) The heart rates were analyzed. (**E**) The EF values were measured. For 5B and 5C, the significance of the difference between groups was determined using one-way ANOVA. For 5D and 5E, the significance of the difference between groups was determined using two-way ANOVA (n = 6/group for the blood pressure tests of the cSD + LTF-Day1 and cSD-Day2 group; n = 10/group for the D and E; ** *p* < 0.01, *** *p* < 0.001). (**F**) Hematoxylin and eosin (H&E) images of crosscut section heart of control, cSD, and cSD + LTF mice (scale bar = 1 mm or 100 μm). (**G**–**M**) Representative western blot image and quantification of LC3-I, LC3-II, p62, Bax, Bcl2, and c-Cas-3 SOD2 in control, cSD, and cSD + LTF mouse hearts (n = 3/group). The significance of the difference between groups was determined using independent *t*-test. (**N**) Volcano map showing upregulated (red) or downregulated (green) genes in both cSD group and cSD + LTF group. Vertical dashed lines denote fold-change thresholds (|log2FC| = 1); the horizontal dashed line denotes the *p* adjust threshold (* *p* < 0.05). (**O**) Heat map of fold-changes between cSD and cSD + LTF. Red indicates a high level of gene expression, and blue indicates a low level of gene expression. (**P**) GO enrichment analysis identified significantly enriched or under-represented biological processes for DEGs of the cSD group and cSD + LTF group. The size of the dot is proportional to the number of differential genes that correspond to the item. The horizontal coordinate represents the ratio of the number of differential genes matched to the item to the total number of differential genes. (**Q**) Venn diagrams comparing DEGs identified in no-cSD, cSD, and cSD + LTF. The Venn diagram shows that 98 genes are common. Venn diagrams show the size of the intersection (number of shared DEGs) between individual differential gene sets, as well as the differential genes specific to each differential gene set. All error bars are s.e.m. (* *p* < 0.05, ** *p* < 0.01, *** *p* < 0.001.).

Our previous studies showed that long-term low-frequencies (LTF) stimulation of neurons could produce long-term inhibition of neurons [46]. Though instant inhibition of PVN^OXT^ neurons could not affect heart rates, we next tested whether the long-term low-frequencies (LTF) stimulation of the PVN^OXT^ neurons could regulate heart rates following cSD. The AAV-OXT-hChR2-mCherry virus was injected into the PVN (Figure 4A). Three weeks following the virus injection, mice were treated with sleep deprivation for seven days. Mice were photostimulated with trains of 473 nm light (1 Hz, 4 ms, 1800 pulses, 7 mW per mm2 at the PVN) for 15 min in the cSD + LTF-Day1 and cSD + LTF-Day2 groups. LTF simulation decreased c-Fos expression in the PVN^OXT^ neurons (Figure S4D).

We then found that the cSD increased the systolic pressures and diastolic pressures (F (4, 37) = 16.12, F (4, 37) = 8.143; both *p* < 0.001, Figure 4B,C). The cSD decreased the heart rates but did not affect the EF values (F (4, 36) = 32.59, F (4, 36) = 3.606; *p* < 0.001, *p* = 0.091; Figure 4D,E). The LTF stimulation could reverse the effect of the cSD on the systolic pressures and diastolic pressures on the first day (F (4, 37) = 16.12, F (4, 37) = 8.143; *p* = 0.014, *p* = 0.013; Figure 4B,C). The LTF stimulation could not reverse the effect of the cSD on the heart rates immediately after the LTF on day 1 but could significantly raise heart rates on day 2 compared with the no-LTF group (F (4, 36) = 32.59, *p* < 0.001, Figure 4D). However, the heart rates of the cSD group without LTF treatment did not recover to normal levels on day 2. There were no significant differences between the EF values of any of the groups (F (4, 36) = 3.606, *p* > 0.05, Figure 4E).

We also found some other interesting phenomena: stimulation of the PVN^OXT^ neurons with different modes of light provoked opposite effects on the heart rates of cSD mice. The cSD decreased heart rates significantly, and optogenetic activation of PVN^OXT^-ChR2 neurons with 20 Hz blue light seriously decreased the heart rates after the cSD, with some subjects showing intermittent cardiac arrest (see Supplementary Video S1 data). Immediately after the 20 Hz blue-light stimulation, the LTF stimulation (1 Hz blue light, 30 min) was delivered to stimulate the PVN^OXT^ -ChR2 neurons. We found that the intermittent cardiac arrest disappeared after the LTF, and the heart rates recovered to the cSD group levels without 20 Hz light stimulation (see Supplementary Video S1 data).

We then tested whether the LTF might minimize heart damage generated by cSD in mice. As shown in Figure 4F, myocardial cells were arranged neatly, with dense cytoplasm and clear contour boundaries in the normal group of mice. However, cSD altered the morphological structure of myocardial cells, leading to disordered arrangement, less dense cytoplasm with granular degeneration, and unclear contour boundaries. In comparison to the cSD group, the proportion of damaged myocardial cells was significantly reduced in the cSD + LTF group (Figure 4F). Interestingly, in mice, cSD + LTF reduced the effects of cSD on the expression of proteins related to autophagy and apoptosis (Figure 4G–M). Reduced protein levels of LC3-II and LC3-II/LC3-I ratio (t(4) = 14.60, *p* = 0.001; t(4) = 3.293, *p* = 0.0301; Figure 4G–I), reduced expression of Bax and Cleaved Caspase-3 (t(4) = 4.661, *p* = 0.0096; t(4) = 6.933, *p* = 0.0023; Figure 4G,K,M), and increased expression of p62 (t(4) = 10.57, *p* = 0.0005; Figure 4G,J) and Bcl2 (t(4) = 4.452, *p* = 0.0112; Figure 4G,L) were found. These findings showed that LTF might shield cardiac tissue against excessive autophagy and apoptosis.

Secondly, we sought to investigate the impact of long-term low-frequencies (LTF) stimulation on this transcriptional signature in PVN^OXT^ neurons. The largest number of differentially expressed genes, 400, was detected when PVN^OXT^ was stimulated with LTF after sleep deprivation. Of these genes, 16 were upregulated (log2 fold change ≥ 1 and *p* adjust < 0.05), while 384 were downregulated (log2FC ≤ −1 and *p* adjust < 0.05; Figure 4N). Surprisingly, the genes that we found to be significantly downregulated due to LTF stimulation of PVN^OXT^ following chronic sleep deprivation were Atf5, mPST, Mthfd2, and Shmt2 (log2 fold change < −2 and *p* adjust < 0.005; Figure 4O), which were significantly connected with autophagy and cellular metabolism. The LTF stimulation of PVN^OXT^ following cSD impacted a variety of processes, including autophagy (Atf5 and Rhd), protein catabolism (Ptdss2, Dusp8, and Rnf114), oxidative stress (Ccs and Eif4ebp1), and regulation of numerous critical biological functions, according to functional enrichment analysis for these 400 differentially expressed genes (*p* adjust < 0.0001; Figure 4P). In total, 98 differentially expressed genes were common between the two categories (Figure 4Q). These results indicate that LTF stimulation of PVN^OXT^ following cSD reduced cellular stress responses. Thus, the results from RNA-Seq analysis in blood validated the protective role of LTF stimulation of PVN^OXT^ in the cardiovascular system after chronic sleep deprivation. We also found that cSD decreased the OXT level (Figure S5). LTF could further decrease serum OXT levels (Figure S5). These results suggested that the decrease of OXT level by LTF simulation had a protective effect on the cardiovascular system.

### 3.5. The Increased mPST Release from the Peripheral Blood Was Associated with Reduced Excitability of PVN^OXT^ Neurons and Abnormal Cardiovascular Response Following cSD

**Figure 5:**
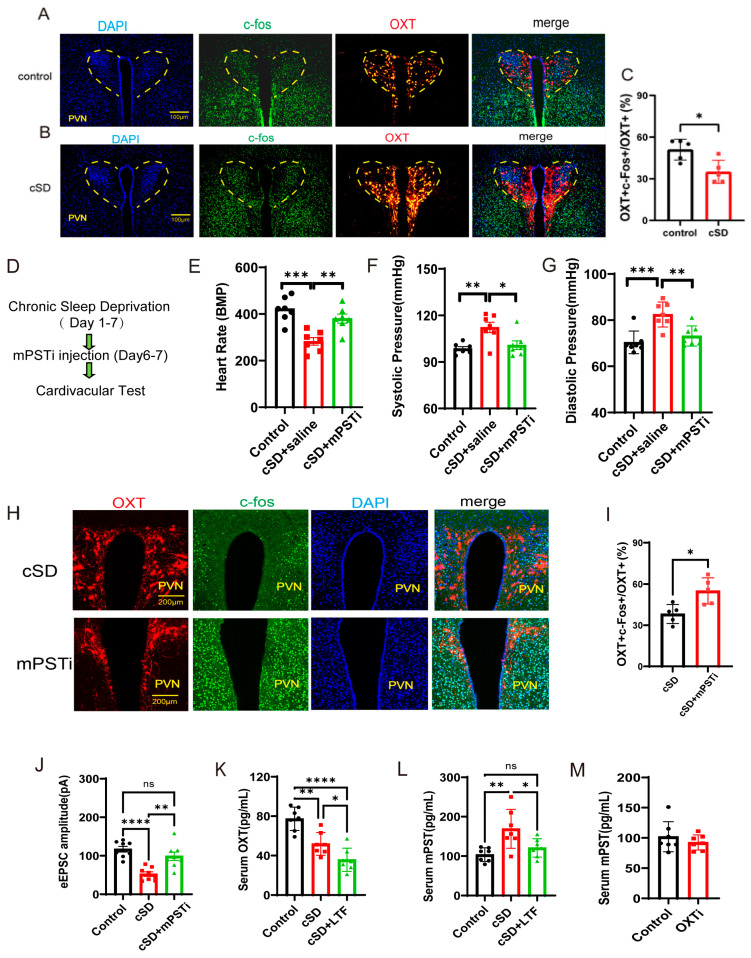
The increased mPST release from the peripheral blood was associated with reduced excitability of PVN^OXT^ neurons and abnormal cardiovascular response following cSD. (**A**,**B**) Co-staining of PVN^OXT^ neurons with OXT antibody (red), c-Fos antibody (green), and DAPI (blue). Scale bars, 100 μm. (**C**) Statistical analysis results of slice pictures taken with confocal fluorescence microscopy showed the percentage of c-Fos-positive neurons in OXT-positive neurons in the control and cSD group (n = 5 mice). (**D**) A schematic diagram of the experiment (mPSTi, mPST inhibitor) (**E**) A comparison of the heart rates of mice between different groups is shown. (**F**,**G**) A comparison of the systolic diastolic pressure of mice between different groups is displayed. (**H**) Co-staining of PVN^OXT^ neurons with OXT antibody (red), c-Fos antibody (green), and DAPI (blue). Scale bars, 200 μm. (**I**) Statistical analysis results of slice pictures taken with confocal fluorescence microscopy showed the percentage of c-Fos-positive neurons in OXT-positive neurons in the cSD and cSD + mPSTi group (n = 5 mice). (**J**) The peak amplitudes of the evoked excitatory postsynaptic currents (eEPSC) in diffirent groups (n = 6 mice). The serum OXT (**K**) and mPST (**L**,**M**) were measured by ELISA kits. Vertical bars represent the mean ± the SEM. Asterisks indicate significant differences from the relevant controls (ns *p* > 0.05, * *p* < 0.05, ** *p* < 0.01, *** *p* < 0.001, **** *p* < 0.0001, independent *t*-test for (**C**, **I**) and (**M**); one-way ANOVA for (**E**–**G**) and (**K**,**L**)).

Quantitative analysis of confocal imaging data revealed a significant reduction in c-Fos/OXT neuronal co-activation indices within the PVN of cSD subjects (Figure 5A–C). These findings demonstrated that cSD induced the attenuation of oxytocinergic neuronal excitability, characterized by diminished neuronal activation thresholds in PVN^OXT^ (Figure 5A–C,J). We then investigated the underlying mechanism. The transcriptome of the blood showed the cSD-upregulated genes were Atf5, mPST, Mthfd2, Slc7a5, Shmt2, and Rexo2, among others (Figure 1I). Among these genes, mPST has attracted our attention because of its previously reported involvement in the regulation of the brain and cardiovascular system [18,19]. We thus hypothesized that cSD-elicited mPST release from the peripheral blood might directly lead to the abnormal cardiovascular response, affecting the neuronal activity of PVN^OXT^ neurons directly or indirectly.

We tested whether mPST was associated with increased anxiety level and abnormal cardiovascular response following cSD. The inhibitor of mPST (mPSTi) and saline was intraperitoneally injected in the cSD + mPSTi group and cSD group, respectively, before the last two-day cSD (twice, one injection/day, 16 mg/kg). The cardiovascular tests and behavioral tests were performed after cSD. We found that the cSD + mPSTi group also showed increased heart rates, decreased systolic pressure, and decreased diastolic pressure compared with the cSD group (F (2, 18) = 14.47, 7.786 or 14.47; *p* = 0.004, 0.017 or 0.004; both *p* < 0.0001; Figure 5E–G). These results suggest that inhibiting mPSTi could reverse cSD-elicited abnormal cardiovascular response following cSD.

To further explore whether mPST could affect the activity of PVN^OXT^ neurons, co-staining of PVN^OXT^ neurons with OXT antibody (red), c-Fos antibody (green), and DAPI (blue) was performed (Figure 5H). Statistical analysis results of slice pictures showed the percentage of c-Fos-positive neurons in OXT-positive neurons in the cSD + mPSTi group was increased compared with that of the cSD group (t (8) = 3.196, *p* = 0.012; Figure 5I). In addition, we also found that the amplitudes of evoked excitatory postsynaptic currents (eEPSCs) were increased in cSD + mPSTi mice compared to the cSD mice (F (2, 21) = 16.21; *p* = 0.002; Figure 5J). Altogether, we inferred that the cSD-increased mPST release from the peripheral blood might decrease the excitability of PVN^OXT^ neurons and induce abnormal cardiovascular response following cSD.

We also found that cSD decreased the OXT level and increased mPST protein in the serum. LTF could further decrease serum OXT levels but reversed the cSD-induced increase of serum mPST (Figure 5K–L). The optogenetic inhibition of PVN^OXT^ in normal mice (OXTi) did not change serum mPST levels (Figure 5M). These results suggest that although the mPST release could inhibit PVN^OXT^ neurons, the long-term decrease of OXT level by LTF simulation in turn has a feedback inhibition effect on the mPST expression, which helps to explain why LTF stimulation of PVN^OXT^ neurons has a protective effect on the cardiovascular system. However, the short-term inhibition of PVN^OXT^ neurons did not affect the mPST expression, which helps to explain why optogenetic inhibition of the PVN^OXT^ neurons had no effect on heart rates, systolic pressures, diastolic pressures, or the EF values of normal mice without cSD treatment.

## 4. Discussion

OXT—the “love hormone”—binds to specific OXT receptors (OXTRs), which were found to functionally couple with both Gq/11 protein and V1aR [47]. It has been reported that OXT synthesized in the heart supports cardiac development, regeneration, and function [48,49] and lessens cardiovascular reactivity to stress [50,51]. Oxytocin has been proposed to be an anti-stress hormone with regard to the cardiovascular axis [51,52]. The PVN^OXT^ neurons are a group of special neuroendocrine cells that not only retain the firing characteristics of neurons but can also release hormones into the blood to regulate the peripheral target organs [53]. The PVN^OXT^ neurons might regulate the cardiovascular system from both the neural circuit and the neuroendocrine pathway. Though we found that optogenetic activation of the PVN^OXT^ neurons could decrease the heart rate, it could not affect the heart rate in the presence of the OXT receptor inhibitor atosiban (which cannot pass the blood–brain barrier, Figure 2K). These results suggested that the PVN^OXT^ neurons might be released in the posterior lobe of the pituitary gland and transported to various target organs through the blood. The released OXT then regulates the heart through the OXT receptors in the peripheral cardiovascular system, consistent with previous findings that used the retrograde viral tracing method to label the PVN oxytocin neurons in the posterior pituitary circuit [54].

We found that increased mPST could directly mediate cSD-induced abnormal peripheral cardiovascular responses. We can infer the following conclusions from our results: The decrease of OXT initiated inhibition of peripheral mPST when both of the following conditions were met: a large increase in mPST caused by the pathologic model (exceeding the threshold amount of mPST) and a long-term decline in OXT release (e.g., LTF-induced). Under normal circumstances, when the expression of mPST is not high (below the threshold), changes in OXT secretion might have little effect on mPST release and mPST-mediated abnormal cardiovascular response. This could also explain why optogenetic inhibition of the PVN^OXT^ neurons had no effect on heart rates, systolic pressures, diastolic pressures, or the EF values of normal mice without cSD treatment.

It was still not clear whether PVN^OXT^ neurons regulated the cardiac system through direct neuronal transmission, or whether the oxytocin neurons in the PVN brain area were the same type of neuron populations that connect to the heart. We then explored whether there was a direct neural loop connection between the PVN^OXT^ neurons and the heart by using the pseudorabies virus for heart-to-brain retrograde labeling. We found that almost all pseudorabies virus retrograde labeling neurons originating from the heart are not OXT-positive neurons in the PVN (~96%) (Supplementary Figure S6). Besides, if it were direct neural regulation, it should be an immediate effect. However, we found a delay in the regulation of heart rates and blood pressure by PVN^OXT^ neurons, which usually acted 3–5 min after the optogenetic activation of PVN^OXT^ neurons. All these studies suggested that PVN^OXT^ neurons regulate the cardiovascular system mainly through the neuroendocrine pathway.

Optogenetic inhibition of the PVN^OXT^ neurons did not affect heart rates, systolic pressures, diastolic pressures, or the EF values of normal mice without cSD treatment. However, short-time inhibition of PVN^OXT^ neurons could quickly restore blood pressure to normal after the cSD. We speculated that the PVN^OXT^ neurons did not participate in the maintenance of blood pressure and heart rates in the normal condition but might be involved in regulating blood pressure and heart rate after the cSD. This conclusion was consistent with the result that cSD reduced the excitability of PVN^OXT^ (Figure 5A–C) and decreased heart rate, which means that cSD-elicited decreased heart rate might not be due to the changed activity of PVN^OXT^ neurons. On the contrary, the decrease in the activity of PVN^OXT^ neurons may be one of the mechanisms by which the body initiates self-protection of cardiovascular activities. Thus, further LTF-mediated inhibition of PVN^OXT^ neurons improved the cardiovascular system following cSD.

We found that LTF stimulation (long-term inhibition) of PVN^OXT^ neurons following cSD could reverse the morphological structure of myocardial cells and the autophagy and apoptosis of cardiac tissue induced by the cSD. To explore the mechanism, we analyzed the transcriptome results of the whole blood. As we know, the whole blood sample was collected for transcriptome analysis. Blood is mainly composed of plasma, blood cells (red blood cells, white blood cells), and platelets. Platelet cells are anucleate cells and contain approximately 2.2 femtograms of total RNA, nearly 4 times more than their larger anucleate cell lineage, the erythrocytes, but approximately 1000 times less than nucleated cells (white blood cells) such as T cells, B cells, or granulocytes [55,56]. Consequently, the transcriptional profile of peripheral blood predominantly reflects leukocyte activity, as these immunocompetent cells constitute the primary mediators of host defense mechanisms against pathogenic invasions and multifactorial disease progression.

The transcriptome results of the whole blood showed that cSD resulted in 114 genes that were differentially expressed (105 upregulated and 9 downregulated), showing the over-activated immune system in the blood. LTF stimulation of PVN^OXT^ neurons upregulated 16 genes and downregulated 384 genes following cSD, suggesting inhibited immune activity in the blood. The genes that were significantly downregulated due to LTF stimulation of PVN^OXT^ neurons following cSD were Atf5, mPST, Mthfd2, and Shmt2 (Figure 4), which were significantly connected with autophagy and cellular metabolism. LTF stimulation of PVN^OXT^ following cSD improved a variety of processes, including autophagy (Atf5 and Rhd), protein catabolism (Ptdss2, Dusp8, and Rnf114), and cellular oxidative stress (Ccs and Eif4ebp1). Consistent with our results, another lab found that after 4 whole days of sleep deprivation, mice exhibited severe inflammation, and approximately 80% died [37]. Mechanistic analysis demonstrated chronic SD elevates cerebral prostaglandin D2 (PGD2) through compromised blood–brain barrier permeability, provoking neutrophil infiltration and cytokine-storm-like syndrome activation via peripheral circulation [37]. Among these genes, mPST has attracted our attention because of its previously reported involvement in the regulation of the cardiovascular system. Previous studies have found that CTH/mPST double ablation enhanced vasorelaxation and reduced blood pressure [33]. cSD-increased mPST release from the peripheral blood might enter the heart and induce abnormal cardiovascular response following cSD.

Previous studies have found that long-time inflammation could be detrimental to the heart [57]. We thus speculate that cSD-induced disorder of the immune system and increased mPST release could lead to abnormal cardiovascular responses, such as morphological structure change of myocardial cells and autophagy and apoptosis of cardiac tissue. LTF stimulation of PVN^OXT^ neurons could reverse the cSD-induced increase of mPST release, thus improving the morphological structure of myocardial cells and the autophagy and apoptosis of cardiac tissue induced by the cSD. Our research will help attract more similar studies and provide foundations for researching cSD-induced cardiovascular diseases.

The oxytocin receptors (OXTRs) system exhibits ubiquitous distribution in immune cells and organs, such as rat thymic epithelial cells [58] and bone marrow stem cells [59]. Importantly, the expression of OXTRs in immune tissues can be inducible, which has been shown in bovine peripheral blood mononuclear cells and T lymphocytes [60] and rat mesenchymal stem cells [59]. These findings establish OXT as a potent immunomodulator capable of real-time cellular adjustments through receptor-mediated signaling, enabling precise adaptation to immunological stressors [61]. Oxytocin neurons can integrate information from presynaptic neurons or sense concentrations of blood-borne substances such as inflammatory factors [57], and in turn secrete an appropriate amount of oxytocin into the blood and brain. It could preset the immune system in an optimal working condition by regulating the activity of bone marrow, thymus, and T-/B-cells, as well as other immune organs and tissues [57], thereby maintaining the homeostasis of immune functions and inhibiting immune damage. However, oxytocin could worsen immune injury in parturient women with latex allergy and bronchial asthma [62], chorioamnionitis [63], and premature birth [64]. The mechanism of LTF stimulation of PVN^OXT^ neurons on the immune system of mice after cSD was complex. We injected the AAV-OXT-ChR2-mCherry virus into the PVN and performed optogenetic inhibition of OXT neurons with 30 min blue-light stimulation (1 Hz, 473 nm) 3 weeks following virus expression. As we know, under blue-light irradiation, the all-trans-retinal cofactor absorbs photons and undergoes conformational changes, causing the ChR2 channel to open, causing Na+ inflow, and leading to cell membrane depolarization and cell activation. Therefore, in the early stage of 30 min of stimulation, even with 1 Hz stimulation, neurons were excited at a low frequency, causing the release of oxytocin. However, as the duration of this low frequency increases, the neurons underwent the opposite change, and long-term excitability suppression and oxytocin release were inhibited. We also found that the activity of PVN^OXT^ neurons was inhibited by the LTF stimulation. The comprehensive effect of the LTF process was protective for the immune system in the cardiovascular system following cSD.

Vasopressin and oxytocin, two neurohypophysial peptides, are now known to regulate the cardiovascular system from both the brain and its peripheral components (the kidneys, vasculature, and heart). Emerging research over the past decade has elucidated novel neuroendocrine regulatory pathways governing the secretion of vasopressin and oxytocin: autocrine and paracrine factors, neuronal transmission, and neuroinflammation. These novel discoveries indicate prospective targets in hypertension and heart failure for creating new, centrally acting cardiovascular medicines. Prior investigations utilizing pressure-overload-induced cardiac insufficiency models have established that targeted stimulation of oxytocinergic neurons significantly improves myocardial contractility parameters [65]. In rats, hypertension brought on by prolonged intermittent hypoxia/hypercapnia was avoided by activating oxytocin neurons [27]. Interestingly, we found the opposite function in the cardiovascular system under the cSD model. We discovered that chronic sleep deprivation decreased heart rates and increased blood pressure in mice. Instant optogenetic activation of PVN^OXT^ neurons immediately after the cSD further worsened the cardiovascular responses. Long-term inhibition of PVN oxytocin neurons could promote the recovery of decreased heart rates and elevated blood pressure induced by cSD. We speculated that central oxytocin neurons’ roles differed in different pathological models and could not be simply defined as protective or damaging. For example, in chronic intermittent hypoxia/hypercapnia model [48], decreased heart rates induced by the oxytocin neurons could help animals to reduce oxygen consumption, thus playing a protective role in the cardiovascular system. However, PVN^OXT^-mediated reduced heart rates could further worsen the cSD-elicited suppression of heart rates and abnormal cardiovascular responses. Although our study revealed the role of oxytocin in the effects of sleep deprivation on cardiovascular function, there actually existed multiple neurotransmitter and hormone changes after sleep deprivation, such as catecholamines [66,67,68], indicting that cSD-induced cardiovascular disease may be a complex pathological phenomenon caused by multiple factors.

Epidemiological evidence has highlighted circadian rhythm disruptions as risk factors for multiple diseases, demonstrating significant associations with myocardial infarction, ischemic cardiomyopathy progression, and generalized anxiety pathophysiology. Many neurons with neuroendocrine functions, such as oxytocin neurons, are essential for both the cardiovascular system and anxiety emotions. When previous researchers studied the functions of these neurons, they often only focused on their effects on the central nervous system or only on their effects on the periphery, while ignoring all systemic effects. This paper suggested that while intervening on oxytocin neurons in the PVN, attention should also be paid to the possible side effects on the peripheral cardiovascular system, thereby establishing a foundation for future translational research exploring OXT as a therapeutic intervention to alleviate cardiovascular damage in patients or mice with sleep disorders. Targeting the PVN-OXT circuit pharmacologically represents a promising therapeutic strategy for managing sleep-disordered patients with associated cardiovascular responses.

As we know, synaptic plasticity is associated with learning and memory. Few articles have examined the relationship between neuronal synaptic plasticity and cardiovascular system function. This paper may provide a new direction for the treatment of cardiovascular diseases by regulating the function of the nervous system through synaptic plasticity. Our study introduces an optogenetic approach to examine the molecular mechanisms linking cSD and cardiovascular dysfunction, by specifically targeting oxytocin neurons—a method underexplored in prior sleep/cardiovascular research. While existing studies have primarily focused on systemic factors such as hypoxia or inflammatory cascades in CSD-induced cardiovascular risks, our work demonstrates that optogenetic modulation of oxytocin neurons significantly influences cardiac autonomic balance and vascular reactivity.

While our findings establish a preclinical foundation for targeting OXT neurons in sleep-deprivation-induced cardiovascular dysregulation, specific methods for clinical translation remain unknown. A previous study showed that intranasal OXT significantly increased plasma OXT concentrations within 40 min [69]; thus, we supposed that intranasal oxytocin—a method already proven safe and used for many years [70,71]—could probably be used to modulate cardiovascular disease in patients with insomnia. However, potential interspecies differences should be acknowledged, and dose–response studies in non-human primates are necessary before clinical experiments. Furthermore, the integration of well-established manufacturing technologies with multidisciplinary expertise in optics and electrical engineering serves as a critical technical prerequisite for neural implants to achieve reliable performance in stimulation applications [72]. Besides, our study had a limitation: the AAV vectors used in this study may transfect non-neuronal cell populations, which may decrease the reliability of our work. Thus, in our future work, we would like to use transgenic mice to improve specificity.

## 5. Conclusions

Our study revealed that cSD changed the blood transcriptome, decreased the heart rate, increased blood pressure, and enhanced the autophagy and apoptosis of cardiac tissue. We also found that PVN^OXT^ neurons might not be essential for maintaining heart rate and blood pressure, but these neurons demonstrated significant involvement in cSD-evoked pathological cardiovascular responses. LTF inhibited PVN^OXT^ neurons and improved the change of cardiovascular responses induced by cSD. LTF stimulation of PVN^OXT^ neurons reversed the abnormal transcriptome of the blood, the decreased heart rate, the increased blood pressure, and the autophagy and apoptosis of cardiac tissue elicited by cSD. Our research provided potential targets for abnormal cardiovascular system changes elicited by cSD and might inspire the development of novel treatments for cardiovascular diseases involving deep brain stimulation to induce plasticity at relevant brain areas.

## Data Availability

All materials are available from the corresponding author upon reasonable request.

## Supplementary Materials

The following supporting information can be downloaded at: https://www.mdpi.com/article/10.3390/cimb47040220/s1, Figure S1: Activation of arginine vasopressin neurons (AVP) did not affect the heart rates, but increased both the systolic pressure and the diastolic pressure, which indirectly proved the specificity of the AAV9-OXT-ChR2-mCherry virus.; Figure S2: Optogenetic activation of PVNOXT terminals in the mPFC did not affect the heart rate, blood pressure or left ventricular function.; Figure S3: Instant optogenetic inhibition of PVNOXT neurons did not affect the blood pressure and heart rates of normal mice.; Figure S4: Optogenetic simulation of PVNOXT neurons with 20HZ blue light increased c-Fos expression in the PVNOXT neurons, and the LTF simulation decreased c-Fos expression in the PVNOXT neurons.; Figure S5: cSD decreased the OXT level and LTF could further decrease serum OXT levels.; Video S1: Optogenetic activation of PVNOXT-ChR2 neurons with 20 Hz blue light seriously de-creased the heart rates after the cSD. Figure S6: Almost all PRV retrograde labeling neurons originating from the heart are not OXT-positive neurons in the PVN.

## Abbreviations

The following abbreviations are used in this manuscript:

cSD	chronic sleep deprivation
PVN	paraventricular nucleus
OXT	oxytocin
LTF	long-term low-frequencies
CIH	chronic intermittent hypoxia
LVIDd	left ventricular internal diameter during diastole
LVIDs	left ventricular internal diameter during systole
EF	ejection fraction
FS	fractional shortening
DEGs	differentially expressed genes
OXTi	inhibition of OXT neurons
OXTRs	OXT receptors
