# Supplementary material for: The Role of Oxytocin Neurons in the Paraventricular Nucleus in Chronic-Sleep-Deprivation-Mediated Abnormal Cardiovascular Responses
Source: Curr Issues Mol Biol. 2025 Mar 25;47(4):220. doi: 10.3390/cimb47040220 (PMC12025970; doi:10.3390/cimb47040220)
Supplement: Supplementary file 1 [file cimb-47-00220-s001.zip › Supplementary.pdf]

Supplementary Method part

ELISA

To assess the serum protein levels, 1 ml of trunk blood (eyeball enucleation) was obtained at the end of the study using heparin-coated tubes, and the samples were taken and stored at -80°C. Oxytocin (ab133050, Abcam, Cambridge, UK) and S100β (Elabscience, Wuhan, CN) ere measured by ELISA kits.

Supplementary Data

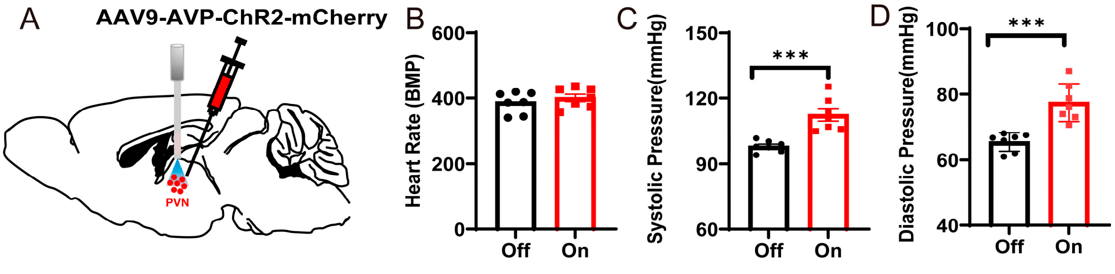

Figure S1. To further determine the specificity of AAV9-OXT-ChR2-mCherry expression in the oxytocin neurons, we injected the AAV-AVP-hChR2-mCherry virus into the PVN. Unlike the functions of oxytocin neurons, activation of arginine vasopressin neurons (AVP) didn't affect the heart rates, but increased both the systolic pressure and the diastolic pressure, which indirectly proved the specificity of the AAV9-OXT-ChR2-mCherry virus. (A) Schematic pictures of AAV-AVP-hChR2-mCherry virus injection and optogenetic stimulation of AVP neurons with 473nm laser.

(B) The heart rates before and during the light stimulation were analyzed.

(C, D) The blood pressures were measured from mice before and during the light-on phase. (n=7/group). Paired t-test. All error bars are s.e.m. ***$p < 0.001$.

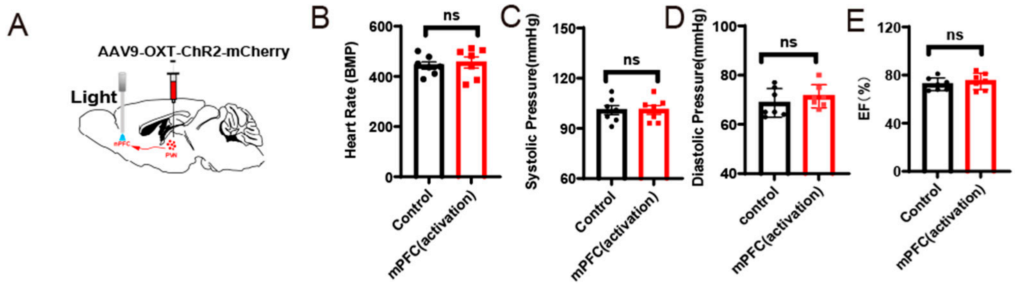

Figure S2. Optogenetic activation of PVN^{OXT} terminals in the mPFC didn't affect the heart rate, blood pressure or left ventricular function.

(A) Schematic illustration of the experiments. For the control group, the AAV9-OXT-mCherry virus was injected into the PVN, and the 473nm laser was on during the behavioral test. For the other three groups, the AAV-OXT-ChR2-mCherry virus was injected into the PVN area. The 473nm laser was delivered into the mPFC for the mPFC (activation) group. The heart rates and EF were measured using Echocardiography.

(B) The comparison of the heart rates of mice between the control and mPFC (activation) group was shown.

(C, D) Blood pressure was measured through the carotid artery in vivo using an animal flowmeter. The comparison of the systolic pressures and diastolic pressure of mice between the control and the mPFC (activation) group was displayed. The comparison of EF(E) between the control and the mPFC (activation) group were separately calculated. No differences were observed between the control group and the mPFC (activation) group. The statistical significance was determined using the independent t-test (n=8/group). All error bars are s.e.m. *$p < 0.05$, **$p < 0.01$, ***$p < 0.001$.

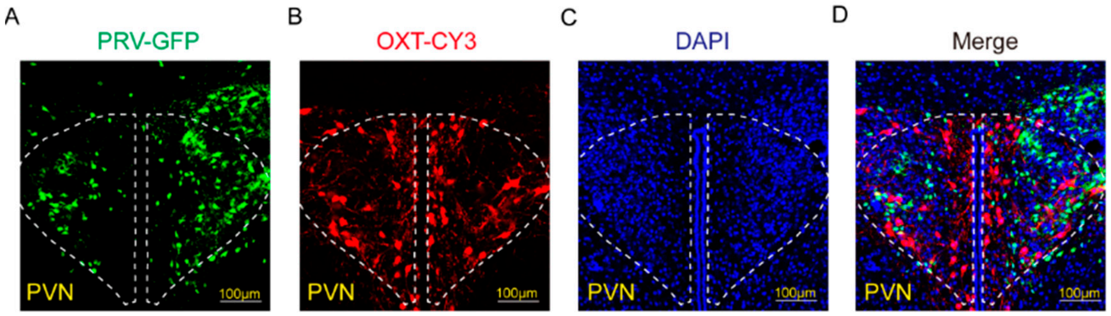

Figure S3. To explore whether there was a direct neural loop connection between the PVN^{OXT} neurons and the heart by using the pseudorabies virus (PRV) for heart-to-brain

retrograde labeling. Almost all PRV retrograde labeling neurons originating from the heart are not OXT-positive neurons in the PVN (~96%).

(A) DAPI staining (blue) in the PVN (blue).

(B) Representative pictures of PRV retrograde labeling PVN neurons originating from the heart(green).

(C) Representative immunohistochemical staining pictures of PVN^{OXT} neuron(red).

(D) Merge images. These pictures were taken with a confocal microscope.

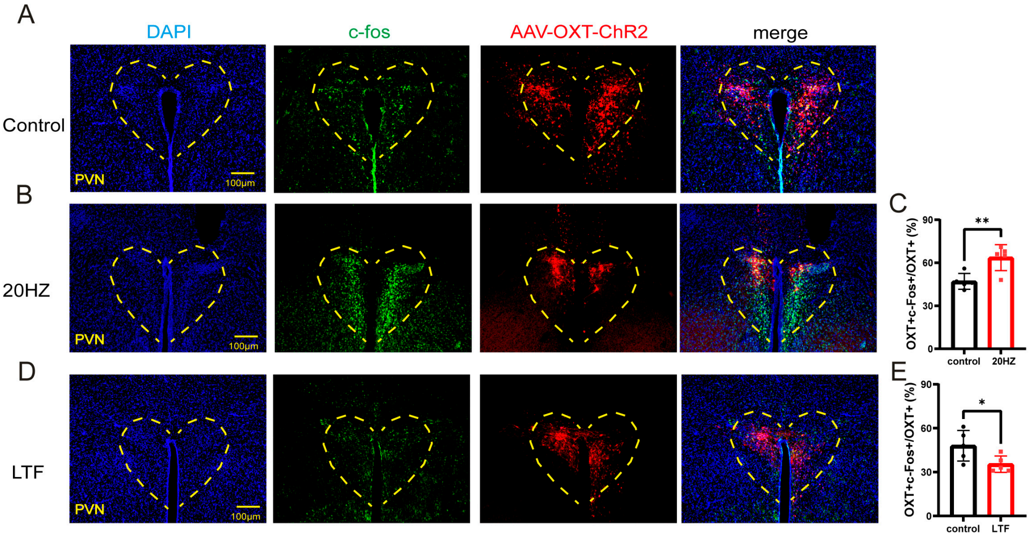

Figure S4. Optogenetic simulation of PVN^{OXT} neurons with 20HZ blue light increased c-Fos expression in the PVN^{OXT} neurons, and the LTF simulation decreased c-Fos expression in the PVN^{OXT} neurons.

(A) Co-staining of c-Fos with AAV-OXT-ChR2-mCherry-expressing neurons without the optogenetic stimulation.

(B) Co-staining of c-Fos with AAV-OXT-ChR2-mCherry-expressing neurons with 20HZ blue light stimulation. Sections of PVN were prepared from adult mice which were transfected with AAV-OXT-ChR2-mCherry. These sections were stained for c-Fos (green) and DAPI (blue). Scale bars, 100μm.

(C) Statistical analysis results of slice pictures showed an increased percentage of OXT-positive neurons in OXT-positive neurons in the 20HZ blue light group.

(D) Co-staining of c-Fos with AAV-OXT-ChR2-mCherry-expressing neurons with LTF stimulation.

(E) Statistical analysis results of slice pictures showed a decreased percentage of OXT-positive neurons in OXT-positive neurons in the LTF group.

The statistical significance was determined using the independent t-test (n=5/group). All error bars are s.e.m. *$p < 0.05$, **$p < 0.01$, ***$p < 0.001$.

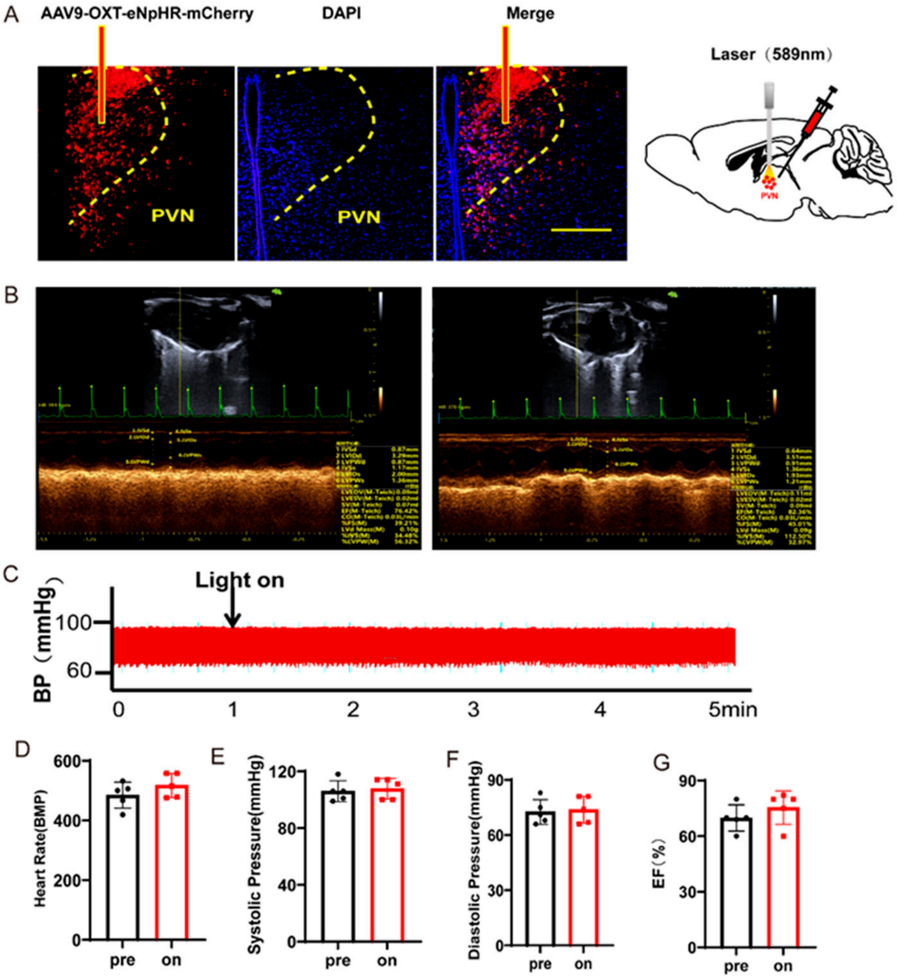

Figure S5. Instant optogenetic inhibition of PVN^{OXT} neurons did not affect the blood pressure and heart rates of normal mice.

(**A**) Representative immunohistochemical staining pictures of AAV-OXT-eNpHR-mCherry viral injection (left panel) and optogenetic inhibition of OXT neurons with 589 nm laser (right panel). AAV-OXT-eNpHR-mCherry expression (red) and DAPI staining (blue) in the PVN were shown. (**B**) Echocardiography results before and after light were shown. (**C**) The representative blood pressure before and during the light-on phase was shown. (**D**) The heart rates before and after the light were analyzed. (**E**) The averaged systolic pressure before and after the light was measured. (**F**) The averaged

diastolic pressure before and after the light was analyzed. (**G**) The EF values were measured before and after the light. The significance of difference between groups was determined using two-way ANOVA (n=5/group). The statistical significance was determined using paired t-test. All error bars are s.e.m.

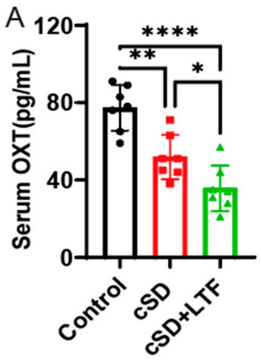

Figure S6. cSD decreased the OXT level and LTF could further decrease serum OXT levels.
(A) The serum OXT were measured by ELISA kits.
